# 

**Published:** 1888-08

**Authors:** 


					﻿Enteredl,dlf,lm,,;.;[.y[;;p[.gkkmkominhbvgyvgbjm,l,,..
Vol. IV.
AUGUST, 1888.
No. 2.
DANIEL'S
MEDICAL
JOURNAL
-E/.DAÑIEb; M. D., Editor, and PuHÌ’»ber,/AUS^V^.\'l>XAS<
<;\- .:3' '<'-. ‘d-- ■■ d «:*	1 ?-< ^ ■>■ •• j- ■ z - A- x
				

